# Volatility and the Cross-Section of Real Estate Equity Returns during Covid-19

**DOI:** 10.1007/s11146-021-09840-6

**Published:** 2021-04-30

**Authors:** Stanimira Milcheva

**Affiliations:** Bartlett Faculty, University College London, 1-19 Torrington Place, London, WC1E 7HB UK

**Keywords:** Covid-19 risk factor, Pandemic, Factor model, Fama–MacBeth, REITs, G1, G14, I1, D84, R30

## Abstract

This paper uses the global systemic shock associated with the outbreak of the novel coronavirus Covid-19 to assess the risk-return relationship in the cross-section of real estate equities in the US and in selected Asian countries. I construct regional Covid-19 Risk Factors (CRFs) to assess how the risk exposure of stocks to the pandemic affects their performance. I find substantial differences between stocks in Asia and the US as a result of the pandemic. During the early stages of the pandemic, the sensitivity of Asian real estate companies to the market becomes negative, while it remains positive and increases in the US. Real estate sectors experience strong divergence in performance in the US while little sectoral difference is observed in Asia. The most affected sectors in the US are retail and hotels, while in Asia it is office. A Fama–MacBeth regression shows evidence for a low-risk effect during the Covid period: while insignificant prior to the pandemic, the return-risk relationship becomes significantly negative during the Covid period, with valuation effects driving the results in both regions. Firms in the US perform significantly worse if their exposure to the pandemic is higher, which is not the case in Asia. The results point towards strong divergence of expectations between US and Asian real estate companies in the onset of Covid-19, which may be associated with the level of prior experience to similar pandemics.

## Introduction

The novel coronavirus Covid-19 emerged in China in December 2019 and was declared a global pandemic on 11^th^ March 2020 by the World Health Organisation (WHO). The virus is highly infectious^1^ but with a lower mortality rate than other coronaviruses such as SARS and MERS.^2^ Given it is a new virus, there has not been a vaccine or a treatment for it at first to prevent it from spreading. Covid-19 has led to the shut-down of entire economies and the paralysis of air traffic around the world. A large number of countries entered into lockdowns and households around the world were asked to stay at home for a large part of the first half of 2020 to prevent the spread of the virus. Only essential businesses were operating during the lockdown periods. China was the first country to impose a lockdown in January 2020, and the first one to open back its economy, in March 2020, while a large number of other countries entered into a series of lockdowns starting about two months later, in March 2020.^3^ During the first couple of months of the coronavirus outbreak, a number of Asian countries such as Singapore, South Korea, Taiwan did not impose lockdowns but instead employed rigorous testing and tracking of the infected cases. Their response was different from those of most other countries due to their previous experience with similar coronavirus outbreaks. The outbreak of Covid-19 has caught most countries without previous experience with similar coronaviruses by surprise and some were slower to react than others.

The economic implications of Covid-19 are expected to be worse than that of the Global Financial Crisis (GFC). According to OECD data, world Gross Domestic Product (GDP) fell about 4.16% in 2020. GDP in the US fell by 3.7% while China is an outlier to report positive growth. It grew at 1.78% in 2020, as compared to 6.1% in 2019. However, before any economic data became available, stock markets were the first to respond to the pandemic and the rise in the Covid-19 infected cases. Asset prices of frequently traded assets contain information about expectations of investors about future cash flows and risks (Harvey, 1989).

While first indications about a new coronavirus emerged on the last day of 2019, stock markets started to factor in potential negative effects associated with the novel coronavirus mostly in late January where the lockdown of the entire China was imposed. The first expectations were that the virus would be contained within China and only companies with supply chains linked to China were initially affected. 21 February 2020 marks the day when stocks internationally started a series of declines adjusting expectations that Covid-19 can spread beyond China as information on multiple infected cases outside China emerged. Around that time, South Korea and Iran reported a large surge in infections. By the end of February, fears about a global outbreak started to materialise. Italy became the first European country to be seriously affected, with the coronavirus spreading fast out of Italy into other European countries. Within a matter of days, stock markets have fallen by up to 40% from their values in the beginning of 2020.

This paper looks into how market expectations changed and risk was re-assessed as a result of Covid-19 for one of the most affected sectors during the early stages of the virus outbreak – real estate. Real estate is among the industries hardest hit as countries around the world have entered in lockdowns and shut most service-related industries. The worst affected sectors can be expected to be the leisure industry such as shops, cinemas, pubs, restaurants, hotels, casinos, etc. Other real estate sectors, such as industrial may be less affected. Having a close look at an industry that is directly affected by the coronavirus outbreak sets the low boundary of the Covid-19 effect on stock valuations as investors would be fast to adjust their expectations for sectors where cash flows and risks are easily identifiable as a result of the pandemic.

In general, real estate companies are characterized by low exposure to market shocks, the dominance of idiosyncratic risks, and the low correlation of real estate stocks with overall equity indices during normal times. The majority of those companies derive more than 80% of their revenues from renting out real estate space. When businesses shut down as a result of lockdown policies, real estate revenues from rents decline in the absence of government subsidies. Market participants therefore can expect a direct effect of Covid-19 associated with a reduction in rental income. Investors may also change their assessment of the riskiness of stocks – the discount rate – which will affect the current valuation of stocks and reflect an increase in the riskiness of certain companies as a result of the pandemic.

The paper looks at how Covid-19 risk exposures affect the cross-section of returns in addition to other systematic and idiosyncratic risks. To identify the effect of Covid-19, I compare the response of real estate company stocks (1) right before and during the early stages of the pandemic, (2) and across two regions – Asian countries and the US. To account for varying exposure of firms to Covid-19 risks I construct regional Covid-19 Risk Factors (CRFs). The CRF is based on daily changes in confirmed coronavirus infections in each region – Asia and the US. Returns are regressed on various measures of risks and other controls in a Fama MacBeth setting in order to assess the cross-sectional link between risk and return. The study looks at sector and regional differences in performance as a result of the pandemic.

To analyse the effect of the pandemic on the economy, early studies have been conducted looking at the stock markets, since they are fastest to response and provide reliable economic data in the early stages of the pandemic. Gormsen and Koijen (2020) show that the Covid-19 outbreak has very similar effects on the stock market response to the effects from the Global Financial Crisis (GFC) in 2008, with sharp declines in stock markets. Alfaro et al. (2020) conduct an event study linking unexpected changes in the trajectory of Covid-19 and SARS to changes in aggregate and firm-level returns by looking at model predictions of anticipated infections. Those models have been used in the early stages of the pandemic and have attracted a lot of general public attention due to very bleak infection forecasts. They find large variation in responses across US companies with responses being stronger for more leveraged firms. They suggest that “debt-laden and capital-intensive firms are less likely to be able to reduce costs as revenues decline”. Ramelli and Wagner (2020) look into the drivers of firm value linked to firm characteristics following the pandemic. They assess how the exposure of US firms to China trade, their debt and cash holdings, affect firm value. The authors find that returns are lower for firms with high trade exposure with China in the early stages of the pandemic but higher since February when the situation in China starts to improve. Furthermore, similar to Alfaro et al. (2020), Ramelli and Wagner (2020) show that more leveraged firms suffer severely during the “Fever” period of the pandemic – from 24 February until 20 March 2020. Similarly, Fahlenbrach et al. (2020) show that firms with less financial flexibility experience lower equity returns up until the announcement of monetary easing by the Federal Reserve on 23 March 2020. Ding et al. (2020) assess the relationship between corporate characteristics and stock price reactions to Covid-19 cases. They also report that firms with strong fundamentals report less strong declines in their stocks. In particular, firms with less debt, more cash and larger profits, in addition to other factors, are behind these reactions. Ling et al. (2020) are the first to look at the effect of Covid-19 on commercial real estate prices. The authors construct a firm-level measure of Covid-19 exposure by using the location of the properties of the real estate companies and linking those with county-level reported coronavirus cases. Regional exposure to Covid-19 based on the property holdings of a firm leads to significant declines in its return on average. However, the effect largely varies by property type. Ling et al. (2020) show that healthcare and technology real estate investment trusts (REITs) observe positive correlation with the firm’s exposure to Covid-19. Retail, office and residential REITs have a negative relationship instead.

In this paper, I build on above research and use how the outbreak of Covid-19 affected the slope of the security market line (SML) for real estate equities. The SML describes the relationship between returns and risks (betas). Classic investment theories suggest that high returns are associated with high risk and low returns with low risk, hence the SML should be upward sloping. However, a number of studies (Black, 1972; Frazzini & Pedersen, 2014; Asness et al., 2020) find evidence of a so-called ‘low-risk effect’ in which assets with higher risk are associated with lower returns. One explanation for the low-risk effect is associated with leverage constraints (Black, 1972; Frazzini & Pedersen, 2014). This includes liquidity considerations (Malkhozov et al., 2016) and benchmark constraints (Brennan, 1993; Baker et al., 2011). The other dominant explanation is associated with idiosyncratic risks and is linked to behavioural biases. Ang et al. (2006, 2009) demonstrate that stocks with low idiosyncratic volatility are associated with high returns attributing it to behavioral biases and sentiment factors. Asness et al. (2020) claim that such biases can be captured by investor’s preference for lottery-like returns (Barberis & Huang, 2008; Brunnermeier et al., 2007).

The study finds that the effect of Covid-19 is associated with steep declines in average daily returns of real estate returns and a steep increase in market and idiosyncratic risks. Although the pandemic originated in China and first spread throughout Asia, returns of Asian-based companies were less negatively affected as compared to those in the US. The two regions differ in the way stocks specialized on individual real estate sectors respond to the pandemic. While US real estate companies show strong differences in performance based on the real estate sector they specialize on during the Covid-19 period, little sectoral variation is observed in the Asian region. Retail is the sector with significant underperformance during the pandemic in the US. This is also the case for the sensitivity to Covid-19 risk exposure. The factor models incorporating the CRF show that hotel has the highest sensitivity to Covid-19 risks, while in Asia and it is the office sector. The sensitivity of firms during the Covid-19 period increases for most risk factors in the US. In Asia, I observe that the sensitivity prior to the pandemic is close to zero and becomes largely negative during the first few months of the coronavirus. This suggests that Asian and US firms have very different response to the pandemic which may be due to the experience with similar coronaviruses in Asia. Asian real estate companies can provide a good hedge during periods of global economic shocks, although their non-market performance is strongly significantly negatively affected during the pandemic.

Fama MacBeth regressions indicate a significantly negative cross-sectional relationship between returns and risks during the Covid-19 period while no significant relationship is reported prior to that. This points towards a low-risk effect triggered by Covid-19 and associated with valuation effects rather that sentiment. High past Covid-19 sensitivity negatively affects returns in the US but not in Asia. Overall, above findings are indicative of divergence of expectations between US and Asian real estate companies in the onset of Covid-19, which may be associated with the level of prior experience to similar pandemics.

## Relevant Literature

The paper relates to the literature looking at idiosyncratic volatility and the cross-section of equity returns. The seminal work of Ang et al. (2009) is one of the first ones to document this relationship. The authors show that idiosyncratic volatility can explain the cross-sectional variation in returns. Counter to the common intuition and classic investment models, the relationship is found to be negative, suggesting that investors demand stocks with high idiosyncratic risk. They do not explain the mechanisms behind it but conclude that the effect is highly significant. A host of papers follow on to explore various channels explaining this puzzle.

Several reasons for the failure of the risk–return trade-off implied by the Capital Asset Pricing theory have been discussed in the literature. Among those are channels associated with (1) leverage constraints (Asness et al., 2014; Black, 1972; Frazzini & Pedersen, 2014; Li, 2016), (2) benchmarked institutional investors (Baker et al., 2011; Brennan & Subrahmanyam, 1995), (3) money illusion (Cohen et al., 2005; Modigliani & Cohn, 1979), (4) disagreement among investors (see, e.g., Hong & Sraer, 2016; Bali et al. 2018), (5) market-wide sentiment-induced mispricing (see, e.g., Shen & Yu, 2012; Asness et al., 2020). A recent study by Bali et al. (2018) argues that the low-risk effect may be due to divergence of opinion among investors. Investor disagreements about the firm value are caused by unusual firm-level news flows. More pessimistic investors may face restrictions on short selling and hence be prevented from expressing their views and those may not be reflected in the price. Therefore, the negative relationship between idiosyncratic volatility and cross-sectional returns reflects optimistic views directly following the news flows. Li (2016) show that high macro-beta stocks yield low returns as compared to low macro-beta stocks as a result of macro disagreements. Rachwalski and Wen (2016) show that stocks with high idiosyncratic volatility earn low subsequent returns for a few months which they explain by temporary underreaction to idiosyncratic risk innovations.

Wang et al. (2017) also test the negative risk-return relationship showing that it holds among firms in which investors face prior losses. It does not hold across firms that experience gains. An explanation for that might be reference-dependent preferences of investors. Another set of studies (Bali et al., 2011, 2014; Barberis & Huang, 2008) consider that investors who prefer returns with high skewness and those firms tend to be high-risk firms and overpriced, hence earning low abnormal returns. Asness et al. (2020) investigate the low-risk effect stipulated in Black et al. (1972) when assets with low risk are associated with high alphas. One reason for that could be leverage constraints and hence risk should be measured using beta. Another could be associated with behavioural effects and hence risk should be measured using the volatility of idiosyncratic risk. The authors introduce two new factors: betting against correlation (BAC) factor for the market risk and a scaled maximum return (SMAX) factor for the idiosyncratic risk in order to disentangle the drivers of the low-risk effect. They find that both factors play a role in explaining the low-risk effect consistent with leverage and lottery theories of this relationship. This paper adds to above literature by examining those relationships in the context of an unexpected global systemic shock associated with Covid-19.

## Methodology

The analysis is conducted in three steps. In step one, I construct a Covid-19 Risk Factor (CRF). In step two, I estimate Fama French factor models for each company for two periods – “Pre”-Covid-19 and a “Covid-19” period. As part of this step, I also estimate factor models including the CRF. The sample period starts however later due to the inclusion of the CRF. The third step consists of estimating Fama–MacBeth cross-sectional regressions for each region and each period.

### Step 1: Covid-19 Risk Factor

To account for risks associated with firm’s exposure to Covid-19, I construct regional CRFs. The construction of the factor follows the standard approach, grouping companies into the top and bottom quartiles of a distribution and creating a long-short portfolio. In this case, I sort firms based on their exposure to Covid-19 reported cases in the respective country. To construct the factor, there are a number of steps.

First, in order to sort firms on their exposure to Covid-19, I estimate a factor model of daily stock returns for each company for the early Covid-19 period – from 24 January until 28 February 2020 – including national Covid-19 reported cases. The factor model is given as:

1 $${r}_{i,r,t}={\alpha }_{i,r}+{\beta }_{1}{MSCIWorld}_{t}+{\beta }_{2}{FTSE\_EPRA\_NAREIT}_{r,t}+{{\beta }_{3}CovidCaseGrowth}_{r,t}+{\varepsilon }_{i,r,t}$$

with ${r}_{i,r,t}$ the daily return for company *i* in region *r* and day *t*. We distinguish between two regions – Asia developed and the US. ${\alpha }_{i,r}$ is the non-market return or alpha. ${MSCIWorld}_{t}$ denotes the MSCI World stock market index based on closing prices in USD. ${FTSE\_EPRA\_NAREIT}_{r,t}$ is the FTSE-EPRA-NAREIT real estate price index for region *r*. ${CovidCaseGrowth}_{r,t}$ denotes the log changes in daily cumulative Covid-19 cases in region *r*. ${\varepsilon }_{i,r,t}$ is the firm residual of the factor model. The coefficient of interest is ${\beta }_{3}$ capturing the sensitivity of a company to Covid-19. It is used to sort stocks into two equally-weighted portfolios – a high-exposure to Covid-19 portfolio and a low-exposure one. The former consists of an equally-weighted average of the top 20^th^-quantile of stocks sorted on the absolute value of their exposure to Covid-19. The latter consists of the bottom 20^th^-quantile of the stocks. The CRF is constructed as a portfolio long on the high-Covid-exposure stocks and short on the low-exposure companies. The CRF essentially captured the performance of real estate stocks with the highest sensitivity to changes in Covid-19 cases in each region.

**Fig. 1:**
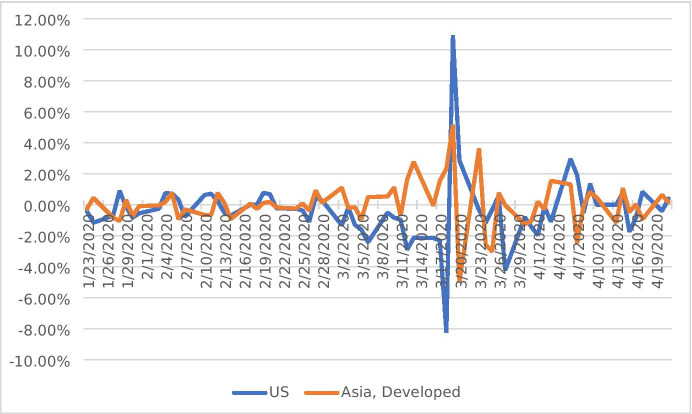
Daily returns of Covid-19 Risk Factors (CRFs). Note: The Covid-19 Risk Factor (CRF) represents a portfolio long on stocks with high exposure to Covid-19 cases and short on stocks with low exposure to Covid-19 cases in the respective regions. The exposure is based on a Factor model with respect to Covid-19 reported infections. The blue line is based on US data for Covid-19 cases and FTSE-EPRA-NAREIT real estate index. The orange line is based on aggregated Covid-19 cases for Japan, Singapore and Hong Kong as well as on the FTSE-EPRA-NAREIT developed Asia index. We also use the Fama French factors for the respective regions. See the text for explanation of the calculation of the CRFs

Figure 1 shows the returns of the CRF for the two regions – Asia developed and the US. As expected, the CRF correctly displays very low volatility prior to the pandemic, as firms should not show systematically different performance based on their exposure to the pandemic before Covid-19 has begun. The CRF becomes volatile during March 2020, which is also the most volatile period on the stock markets during the pandemic. That is when the seriousness of Covid-19 and the implications for the economy started to become clear. We see that while the returns of the CRF in the US have been declining from early March 2020 up until 18 March 2020, the returns of the CRF for Asia developed have mostly remained in positive territory. The two factors seem to mirror each other up until 18 March 2020. The initially negative CRF for the US suggests that stocks with high previous sensitivity to the pandemic substantially underperform stocks with low exposure to it. In the Asian countries Hong Kong, Japan and Singapore, the opposite trend is revealed. Real estate firms with high previous exposure to Covid-19 cases did better in the subsequent period. The CRF in the US displayed a daily return of as low as −8.23% on 18 March 2020 followed by a rise of 11% the following day. The CRFs are a good way to capture the stock market volatility during the early stages of the pandemic, especially during the month of March 2020. This is when lockdown measures and border closures were introduced and where the uncertainty was at its peak. On 16 March 2020, the VIX volatility index capturing uncertainty on financial markets has reached an all-time high. This is also the period where the CFRs show the highest volatility – between 16 and 23 March 2020. Figure 1 also shows that the volatility in the US in that period is higher than in developed Asia – with larger peaks and throughs. It seems that although Asian countries were first hit by the pandemic, their real estate companies have not demonstrated strong divergence in their sensitivity to Covid-19. Instead, US real estate firms have demonstrated large differences in performance associated with their Covid-19 exposure which has resulted in return differentials across companies of up to 11% per day.

Another observation is that real estate firms with higher Covid-19 exposure have not been associated with a long-term value loss but a short-lived re-assessment of their value as the sharp drop in the CRF is followed by a sharp rise in the following period. I assess the effects of the cross-sectional variation to the CRF in more detail in the Results section.

### Step 2: Fama French Factor Models

In the second step, I estimate Fama French factor models for two periods – a “pre”-Covid-19 period (denoted by *P*) and a Covid-19 period (denoted by *C*). The two periods are used for identification purposes. I compare the returns between the two short periods which ensures that everything else is kept constant and the only difference is the pandemic. The *P* period ranges from 1 November 2019 until 22 January 2020. The end date is set by the first reported cases in China and other Asian countries. The *C* period ranges from 23 January until 21 April 2020. 23 January 2020 is when the lockdown of Wuhan province in China was announced and when the news about the new virus started to make headlines internationally. At that time, the World Health Organisation (WHO) had not yet classified Covid-19 as a pandemic and the rest of the world assumed that the virus could be contained within China. Most countries continued with business as usual although some supply chains started to be affected as a by the full lockdown in China a few days after the Wuhan lockdown. While the pandemic has not been reflected to a large extent on the stock markets in the US and most European countries until late February, it was affecting stock markets in Asian countries. Therefore, we use this longer sample as a more conservative measure of the effects of Covid-19 and make the results comparable across continents. The results can be seen as the lower boundary of observable results for the early Covid period which was in February and March 2020. I end the sample period in April as the stock markets and the VIX have stabilised by then with the injection of capital by several central banks, as well as large-scale fiscal measures to boost the economy in response to the pandemic.

For each period, I estimate the following factor model:

2 $${r}_{i,r,t,p}={\alpha }_{i,r,p}+{\beta }_{MF,i,r,p}{MF}_{r,t}+{\beta }_{SMB,i,r,p}{SMB}_{r,t}+{\beta }_{HML,i,r,p}{HML}_{r,t}+{\beta }_{REF,i,r,p}{REF}_{r,t}+{\varepsilon }_{i,r,t,p}$$

with ${r}_{i,r, t,p}$ the daily return for company *i* in in region *r*, day *t* in period *p*. There are two periods, *p* = *P* for the pre-Covid period and *p* = *C* for the Covid period, as explained above. ${\alpha }_{i,r,p}$ is the company alpha. ${MF}_{r,t}$ denotes the Fama French market factor for the respective region; ${SMB}_{r,t}$ denotes the Fama French size factor for the respective region; ${HML}_{r,t}$ denotes the value factor for the respective region; finally, ${REF}_{r,t}$ denotes the FTSE-EPRA-NAREIT real estate factor for the respective region.${\varepsilon }_{i,r,t,p}$ is the firm residual of the factor model. The betas are the coefficients of interest, the risk loadings or factor sensitivities, which will be used as inputs in the third step.

In addition to Eq. (2), I also estimate a different factor model for each firm in each region, this time also including the CRF to account for systematic Covid-19 risks. The model is given as:

3 $${r}_{i,r,t,p}={\alpha }_{i,r,p}+{\beta }_{MF,i,r,p}{MF}_{r,t}+{\beta }_{SMB,i,r,p}{SMB}_{r,t}+{\beta }_{HML,i,r,p}{HML}_{r,t}+{\beta }_{REF,i,r,p}{REF}_{r,t}+{\beta }_{CRF,i,r,p}{CRF}_{r,t}+{\varepsilon }_{i,r,t,p},$$

where ${CRF}_{r,t}$ denotes the Covid-19 Risk Factor for region *r*.

As Eq. (3) incorporates the CRF, I estimate it for a shorter Covid-19 period (denoted as *C2*). The sample period ranges from 28 February 2020 until 21 April 2020. The reason for that is that agents first observe how firms respond to Covid-19 cases and then adjust their expectations and revalue stocks.

### Step 3: Fama McBeth Cross-Sectional Models

The third step consists in estimating Fama–MacBeth cross-sectional regressions for each region and each period. The Fama–MacBeth model is estimated as:

4 $${\overline{r}}_{i,p,r}=a*{\sigma }_{i,p-1,r}+{b}_{f}{*\sum}_{f=1}^N{\beta }_{i,f,p}+d*{Controls}_{i}+f*{Country }_{i}+g*{Sector}_{i}+{\epsilon }_{i,t}$$

with ${\overline{r}}_{i,p}$ the average daily return of company *i* for period *p* with *p* = *P, C, C2*. ${\beta }_{i,f,p}$ are the risk loadings estimated in Eq. (2) for each factor *f*, *f* = *1, …, N* with *N* = 3. ${Controls}_{i}$ include company-specific variables which are mostly related to reported financial data such as the market-to-book (MB) ratio, total asset size, debt-to-assets (DtA) ratio, etc. These variables are reported once a year and would not show variation over the sample period as I only use data from November 2019 until April 2020. I also include country fixed effects and property sector fixed effects. The sector fixed effects are based on the S&P database definition of sector, which will be discussed in more detail in the Data section. Standard errors are clustered by firm ID. ${\sigma }_{i,p-1}$ stays for the past idiosyncratic volatility, or the past residual standard deviation (denoted as *resid SD*) of a firm in *p-1* or the month prior to the beginning of the respective period *p*.

The past idiosyncratic volatility is estimated using the standard deviation of the residuals from the Factor model in Eq. (2) for daily returns for the month preceding the respective period *p*. This is the way past idiosyncratic volatility is estimated in Ang et al. (2009), Bali et al. (2018) and others who look at cross-sectional returns and idiosyncratic volatility shocks.

Finally, I also estimate a Fama-McBeth model including one more explanatory variable – the factor risk loading associated with the CRF, ${\beta }_{i,CRF,p}$, from Eq. (3). The model is given as:

5 $${\overline{r}}_{i,p,r}=a*{\sigma }_{i,p,r}+{b}_{f}{*\sum}_{f=1}^{N}{\beta }_{i,f,p}+c*{\beta }_{i,CRF,p}+d*{Controls}_{i}+f*{Country }_{i}+g*{Sector}_{i}+{\epsilon }_{i,t}.$$

## Data

### Covid-19 Cases

To calculate the CRF, I use information about confirmed Covid-19 infections. The data for daily cases is collated from national sources and provided by John Hopkins University. It accounts for cases that have been tested for coronavirus and confirmed to have the virus. The number of cases does not include all people who may have contracted the virus. In most countries, only people who end up in more serious conditions only or people who are treated in hospitals were tested in the early stages of the pandemic. Each country adopted its own approach to testing with countries like Germany and the Asian countries in our sample conducting much more tests than others, like the UK or the US, in the onset of Covid-19. This was due to lack of testing kits or testing facilities and capabilities. Despite the incomplete way of capturing the exposure to Covid-19, in the early days of the pandemic, the number of confirmed cases was seen as the main source of information about the seriousness of Covid-19. Given the exponential growth in infections, confirmed cases were an indication about how quickly the virus spreads which determines whether or when a country goes in a lockdown. Therefore, increases in the number of confirmed Covid-19 cases could be seen as an indicator of Covid-19 exposure and associated risks from the pandemic. Those risks are systemic in nature as the virus is highly infectious and quickly spreads across countries and communities. As we often associate systemic risks with “contagion”, in the case of Covid-19, the contagion is meant literarily, as evidenced by the fast increase in infected people around the world. Covid-19 risks can therefore be linked to the speed and extent of the contagion of the virus.

### Stock Market Factors

I use the three Fama French factors for each region – US and Asia developed (AD). The three factors are the market factor (MF), the size factor (SMB) and the value factor (HML). Fama and French report factors for the US, Japan, and Asia developed excluding Japan. Therefore, for companies located in the Asia developed region, I use both market factors, for Japan and for Asia developed excluding Japan. To proxy for the real estate market in the respective region, I use the FTSE-EPRA-NAREIT regional indices – for the US and for Asia developed respectively.

**Fig. 2:**
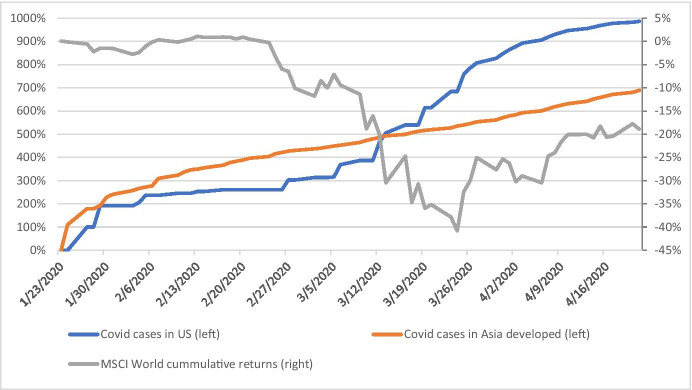
Cumulative daily changes of global stock market returns and Covid-19 cases. Note: Left axis is Covid-19 confirmed cases cumulative growth rate since 23 January 2020. Right axis is MSCI World stock market index cumulative daily returns since 23 January 2020

In the calculation of the CRF, I use a global stock market index – the MSCI World – instead of the Fama and French factors above. Figure 2 plots the MSCI World Index cumulative daily changes between 23 January and 21 April 2020. Prior to 24 March 2020, the relationship between the growth of confirmed cumulative Covid-19 cases and stock market returns is negative (see Fig. 2). This relationship is more pronounced in the US. On 24 March 2020, the stock markets in the US had their “whatever it takes” moment with the Federal Reserve (Fed) announcing that it will do whatever it takes to preserve financial stability. The Fed announced a quantitative easing (QE) program buying a wide range of securities including corporate bonds and commercial mortgage backed securities. Parallel to that, the US and many other countries announced large fiscal support packages to help businesses and households. Once the monetary and fiscal support mechanisms were announced, the equity markets have started to gradually recover the loss in value (see Fig. 2). This is despite a continuation of the growth in Covid-19 cases, as seen in Fig. 2. When we compare Asia and the US, we see that the growth in US Covid-19 cases starts off gradually but towards the end of February sets on a steep increase while Covid-19 cases in Asia follow a steadier but slower increase. By 12 March 2020, the cases in both regions have increased by 500% since the beginning of the pandemic (23 January 2020). The MSCI World index has experienced unprecedented drop falling by 41% as of 24 March 2020 as compared to its value at the onset of the pandemic. The revaluation of companies as a result of Covid-19 news can follow a different pattern in the cross section of firms. The focus of this paper is on the cross-sectional dimension of the Covid-19 shock and on a comparison between real estate company response in Asia and the US. Given that the pandemic has started off in Asia, one can expect Asian-based real estate companies to be harder hit at the onset of the pandemic. However, the experience of the East Asian countries with previous coronaviruses such as SARS may soften the effect of Covid-19, as a result of mechanisms already put in place to respond to a pandemic. Financial markets therefore may incorporate such expectations and regard Asian real estate firms, which have strong local presence, as less affected by the pandemic, as compared to US firms, despite the pandemic hitting the US later in the sample period.

### Company Data

**Fig. 3:**
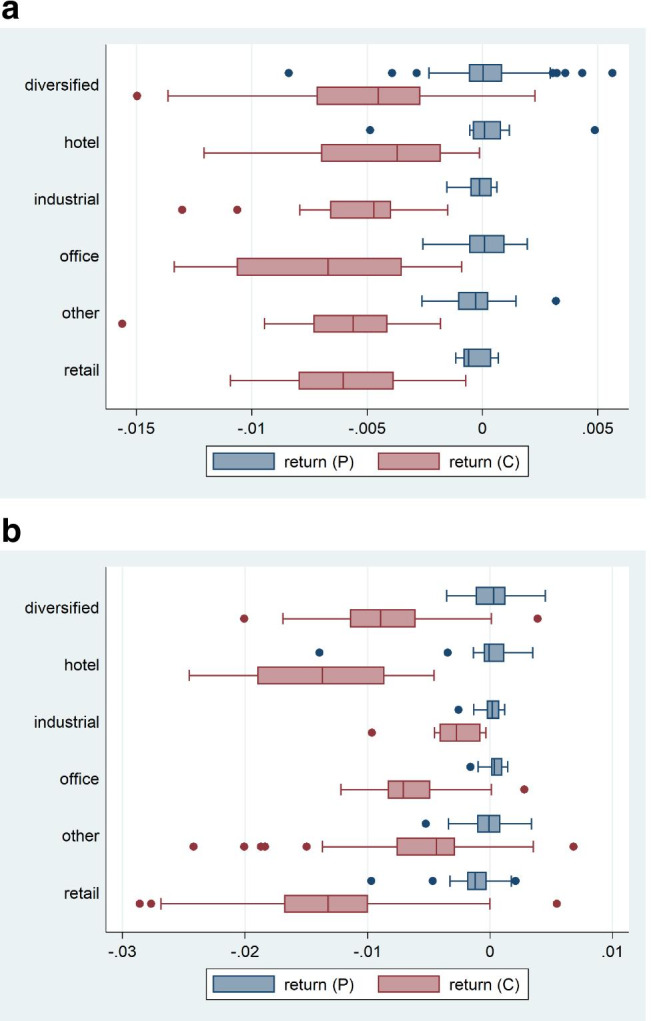
Distribution of daily returns in the cross-section. **a** Asia Developed. **b** US. Note: The box plots show the minimum, first quartile, median, third quartile, and maximum of average cross-sectional returns. The box plots are based on around 200 observations for each region. Asia Developed includes Singapore, Hong Kong and Japan. (P) stays for the pre-pandemic period. (C) for the Covid period. The pre-Covid period is from 1 November 2019 until 22 January 2020. The Covid period is from 24 January 2020 until 21 April 2020

The initial sample contains 447 real estate companies across five countries – US (213), China (36), Hong Kong (66), Japan (74), and Singapore (58). The data comes from S&P Global which was the former SNL Financial. Figure 3 shows the cross-sectional return distribution of the average daily returns in two periods – pre Covid-19 and early Covid-19 – and across sectors. The classification by sector is provided in the database. The majority of the real estate companies have diversified sector portfolio – 186 of the firms in our sample. The second largest group is retail with 53 companies – split between shopping centers, regional malls and other retail. The third and fourth largest sectors are hotel and office with 47 and 46 firms respectively.

The dispersion in returns is much smaller before the outbreak than in the early stages of Covid-19. The dispersion has increased three- to four-fold.

Daily returns are close to zero prior to the pandemic. During the outbreak, the majority of the returns sharply decline and take negative values. During the early stages of pandemic, the average daily return decreases substantially with returns for US hotel as low as −1.5% per day on average. We see that the Asian returns during Covid-19 are on average less negative than the US returns across all sectors.

The left tail of the distribution has shifted considerably further to the left with much lower negative returns in the 25^th^ quantile, below −2.5% for US retail and US hotel sectors. The sector with the largest spread of returns is diversified in Asia and retail in the US. Office has the lowest average return in Asia during the early stages of the pandemic, whereas the most affected sector is hotels in the US.

The results by sectors show large regional differences. The sharp drop in office real estate companies’ stock returns in the early stages of the pandemic can be seen as markets expecting a shift towards more work from home in Asia as compared to the case prior to the pandemic. In the US, the most affected companies are those specializing on retail and hotel which can be interpreted as expecting a the pandemic to lead to a contraction in conventional shopping and consumption as well as less business and leisure travel. Furthermore, this preliminary analysis of the data indicates small difference across sectors in Asia as compared to the US. In the US, the difference in performance across sectors is considerable. This may be seen as a structural shift in the demand for real estate in the US while in Asia it can be seen associated with a decline in economic growth.

Table 1 shows the average daily returns by sector and by region. As in above figures, we see that the returns by sector are much lower during the Covid-19 period. In the US, the lowest daily average return is observed for real estate companies specialising on regional malls (−1.88%), followed by hotels (−1.4%). In Asia, the lowest daily average return is observed for other retail (−0.8%), followed by office (−0.7%). The returns in the US are however twice as low as those in Asia – the Asian market is overall not as badly affected as the US one. The least affected sectors in the US are industrial, self-storage and speciality. As mentioned above, the sectors in Asia do not show large differences in performance and even the least affected sectors are more strongly affected than those in the US.

**Table 1:** Average daily returns by sector and by period for the Asian Region and the US

Asia Developed				US			
Return	Mean	sd	Count	Return	Mean	sd	Count
Casino			0	Casino			3
Pre	NA	NA		Pre	0.1331%	0.1034%	
Covid	NA	NA		Covid	−0.8021%	0.2442%	
Diversified		106		Diversified		37	
Pre	0.0166%	0.1676%		Pre	0.0337%	0.1758%	
Covid	−0.5114%	0.3458%		Covid	−0.8734%	0.4994%	
Health Care		5		Health Care		21	
Pre	−0.0515%	0.0692%		Pre	−0.0607%	0.1806%	
Covid	−0.5874%	0.0618%		Covid	−0.8607%	0.5568%	
Hotel		16		Hotel		28	
Pre	0.0151%	0.1859%		Pre	−0.0066%	0.3102%	
Covid	−0.4434%	0.3467%		Covid	−1.4042%	0.6058%	
Industrial		17		Industrial		13	
Pre	−0.0113%	0.0600%		Pre	0.0019%	0.1047%	
Covid	−0.5565%	0.2917%		Covid	−0.3015%	0.2528%	
Manufactured Home		0		Manufactured Home		3	
Pre	NA	NA		Pre	0.0375%	0.1696%	
Covid	NA	NA		Covid	−0.4494%	0.1608%	
Multifamily		10		Multifamily		12	
Pre	−0.0286%	0.1641%		Pre	−0.0604%	0.0538%	
Covid	−0.6791%	0.4022%		Covid	−0.6080%	0.2959%	
Office		23		Office		21	
Pre	0.0072%	0.1096%		Pre	0.0309%	0.0809%	
Covid	−0.7072%	0.3689%		Covid	−0.6207%	0.3698%	
Other Retail		2		Other Retail		12	
Pre	−0.0814%	0.0033%		Pre	−0.0756%	0.0832%	
Covid	−0.7970%	0.0931%		Covid	−1.1104%	0.6250%	
Regional Mall		8		Regional Mall		7	
Pre	0.0013%	0.0610%		Pre	−0.2698%	0.3265%	
Covid	−0.5630%	0.1663%		Covid	−1.8782%	1.2155%	
Self-Storage		0		Self-Storage		7	
Pre	NA	NA			0.0191%	0.0636%	
Covid	NA	NA			−0.3755%	0.1928%	
Shopping Center		4		Shopping Center		19	
Pre	−0.0799%	0.0286%		Pre	−0.1473%	0.1305%	
Covid	−0.5333%	0.5194%		Covid	−1.3456%	0.5835%	
Specialty		4		Specialty		30	
Pre	0.0021%	0.0974%		Pre	0.0473%	0.1447%	
Covid	−0.4250%	0.1230%		Covid	−0.3648%	0.6134%	

The “Pre” is the pre-Covid-19 period which ranges from 1 November 2019 until 22 January 2020. The “Covid” period ranges from 24 January 2020 until 21 April 2020

The outbreak did not create new losers but exacerbated the problems in already struggling sectors, especially in the retail sector. The biggest changes in performance between the two periods are registered for US companies specializing on hotels and regional malls. The smallest change of returns during Covid-19 is observed for industrial in the US.

Table 2 shows the descriptive statistics of the firm-level variables including the average returns in the pre-Covid-19 and the Covid-19 periods.^4^ In total, the regression analysis includes 179 firms from Asia and 190 firms from the US. The average cross-sectional daily return during the early phase of the pandemic is −0.5% in Asia and −0.86% in the US.

**Table 2:** Descriptive statistics of the firm-level variables

Variable	Obs	Mean	sd	Min	Max
Panel A: Asia Developed					
return Pre	195	0.0000	0.0015	−0.0084	0.0056
return Covid	195	−0.0055	0.0034	−0.0156	0.0023
Total assets	179	61,800,000	163,000,000	19,784	1,210,000,000
DtA	179	0.3575	0.1286	0.0000	0.6519
PtB	179	0.9773	0.4402	0.1086	1.9753
ROAA	180	0.0305	0.0337	−0.2192	0.1244
REIT	199	0.56	0.50	0	1
Panel B: US					
return Pre	213	−0.0002	0.0019	−0.0139	0.0045
return Covid	213	−0.0086	0.0067	−0.0286	0.0068
Total assets	208	6,289,005	7,357,745	17,200	42,800,000
DtA	208	0.4908	0.1843	0.0000	1.3177
PtB	190	2.2626	1.3507	0.5291	7.4319
ROAA	206	0.0337	0.0575	−0.1280	0.4931
REIT	213	0.84	0.37	0	1

DtA stays for the debt-to-assets ratio, PtB stays for the price-to-book ratio, ROAA stays for the return on average assets, REIT dummy takes the value of 1 if the company has a REIT status

The average debt-to-asset (DtA) ratio in 2019 in Asia is lower than in the US. US firms have on average 49% of their assets in debt whereas 36% of the assets in Asian companies is debt. The price-to-book (PtB) ratio is the US is more than twice as high than that in Asian countries, 2.26 as compared to 0.97. One would expect real estate firms to have a PtB ratio of close to one given the nature of the business model of such companies. This is what we observe in Asia however the market in the US seems to have been more heated prior the pandemic with valuations more than twice as high as the book value of the properties. The highest PtB ratio in the US is almost 8 as compared to 2 in China. The cross-sectional variation in market-to-book ratios in the US is therefore much larger. Above observations may also be indicative of the sharper decline in the returns in the US as compared to Asia – given the larger room the adjusting valuations. In terms of performance, both Asia and the US show similar return on assets – around 3–3.3%. About half of the companies in our Asian sample have the real estate investment trust (REIT) status as compared to 84% in the US.

## Results

### Factor Regressions

**Fig. 4:**
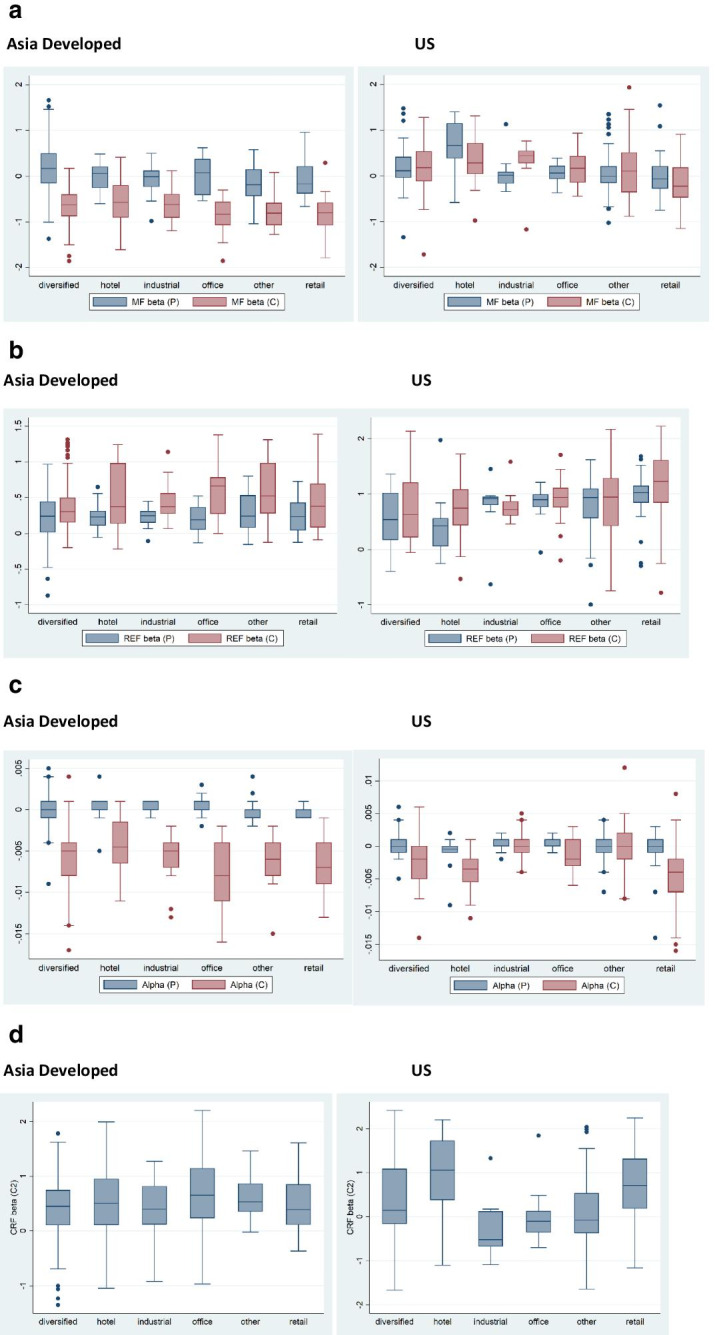
Box plots of cross-sectional betas and alphas before and during the pandemic. **a** Market factor (MF) betas by sector. **b** Real Estate Factor (REF) betas by sector. Note: A box plot shows the minimum, first quartile, median, third quartile, and maximum of our data. The coefficients are estimated using the factor model in Eq. (2) for two periods using listed real estate companies in Hong Kong, Japan, Singapore, and US. The pre-Covid (P) period is from 1 November 2019 until 22 January 2020. The Covid-19 (C) period is from 24 January 2020 until 21 April 2020

The results from the first step, which is based on Eq. (2), are summarised in Table 3. First, I regress each firm on the risk factors to obtain firm estimates. I show the summary statistics of those estimates – alpha and betas – for the pre-Covid and Covid-19 periods. I report the mean, the standard deviation, the 25^th^ and 75^th^ percentile of the distribution. Figure 4 plots the distributions of the coefficients for the two periods.

**Table 3:** Descriptive statistics of listed real estate company daily returns by period

	mean	sd	p25	p75
Panel A: Asia Developed				
Covid-19 Period				
MF beta (C)	−0.7267	0.4543	−0.9810	−0.4520
SMB beta (C)	−0.3919	0.5480	−0.7270	−0.0540
HML beta (C)	−1.5208	0.8599	−2.1240	−0.8450
REF beta (C)	0.4360	0.3711	0.1710	0.6740
Alpha (C)	−0.0060	0.0035	−0.0080	−0.0040
resid SD (C)	0.0096	0.0092	0.0054	0.0111
Pre-Covid Period				
MF beta (P)	0.1000	0.5491	−0.2380	0.3790
SMB beta (P)	0.0770	0.6154	−0.2590	0.3880
HML beta (P)	0.0682	0.4827	−0.1440	0.3470
REF beta (P)	0.2413	0.3222	0.0670	0.4230
Alpha (P)	0.0002	0.0015	−0.0010	0.0010
resid SD (P)	0.0106	0.0062	0.0071	0.0119
Panel B: US				
Covid Period				
MF beta (C)	0.1502	0.5701	−0.265	0.53
SMB beta (C)	0.9028	1.4290	−0.054	1.625
HML beta (C)	0.3391	0.7645	−0.199	0.773
REF beta (C)	0.8966	0.5642	0.49	1.27
Alpha (C)	−0.0018	0.0041	−0.004	0.001
resid SD (C)	0.0086	0.0060	0.0047	0.0094
Pre-Covid Period				
MF beta (P)	0.1729	0.5710	−0.127	0.355
SMB beta (P)	0.2476	0.6137	−0.158	0.569
HML beta (P)	0.1312	0.6414	−0.248	0.417
REF beta (P)	0.7395	0.4700	0.493	1.052
Alpha (P)	−0.0000	0.0019	−0.001	0.001
resid SD (P)	0.0099	0.0062	0.0062	0.0116

The (P) Pre-Covid period ranges from 1 November 2019 until 22 January 2020. The (C) Covid period ranges from 24 January 2020 until 21 April 2020. Betas and alphas are the coefficients associated with Eq. (2). Resid SD stays for the standard deviation of the lagged residual from Eq. (2). The residual is lagged by one month. The Asia Developed sample is based on factor data for developed Asia alone. The US sample is based on factor data for the US alone. MF beta stays for the beta of the region specific Fama French market factor. SMB beta stays for the beta of the region specific Fama French size factor. HML beta stays for the beta of the region specific Fama French value factor. REF beta stays for the beta of the region specific FTSE-EPRA-NAREIT real estate factor. All betas and alphas are estimated using a the factor model in Eq. (2). The residual standard deviation (resid SD) or the past idiosyncratic risk is the SD of the lagged residual

The average cross-sectional market factor (MF) beta before the pandemic is similar in Asia and the US and is close to zero – 0.1 in Asia and 0.17 in the US. The MF distributions between the two regions also seem similar. During the pandemic, we see a divergence in between Asia and the US. The MF beta becomes negative and drops considerably in Asia (−0.73) whereas it remains similar to the pre-Covid period in the US (0.15), however with a change in the distribution and the observation of fat tails. Although the MF beta in the US does not change much during the early stages of the pandemic, the remaining risk factor loadings increase. The largest change is observed in the size factor loading – from 0.25 pre-Covid to 0.9 during Covid-19. The value factor beta also goes up as does the real estate factor loading. In China, betas for all factors, apart from the real estate factor loading, become negative. The size factor and value factor loadings go from almost zero pre-Covid to −0.39 and −1.52 during the pandemic respectively. The real estate beta increases from 0.24 to 0.44.

Asian and US companies demonstrate very different response to market factors as a result of the pandemic. While the US companies become more sensitive to market shocks than before Covid-19, in China companies respond to market volatility counter-cyclically during the outbreak. The huge change in the response to the value factor in Asia from close to zero to more than −1.5 indicates that the pandemic has particularly affected real estate companies through valuation channels. Asian real estate firms negatively overreact to differences in the returns of companies with high book-to-market ratios (value stocks) and companies with low book-to-market values (growth stocks). If the difference in returns of value stocks and growth stocks increases by 1 percentage point, the returns of Asian real estate companies will decrease by 1.5 percentage points on average. For the returns of our firms to be positive, growth stocks should outperform value stocks.

The findings that Asian real estate firms have negative betas in times of a negative economic shock implies that they may provide a good hedge in downturns. In the US we observe what has been documented in previous studies – that listed real estate companies increase their sensitivity to the market in downturns.

Table 3 also shows that the non-market return or alpha turns from close to zero prior to the pandemic to −0.6% and −0.18% per day on average in China and the US respectively during the Covid-19 period. While responding negatively to changes in stock markets, Asian real estate companies deliver strong non-market underperformance during the pandemic. Therefore, it seems that while real estate stocks in Asia respond counter-cyclical to the pandemic, they are still negatively affected due to poor skill (negative alpha). The asset-specific risk (residSD) does not increase during the pandemic suggesting that the increase in the overall stock volatility is driven primary by increased exposure to the market.

Figure 4 shows the distribution of the factor loadings across the US and Asia and across sectors. Panel A shows the betas associated with the market factor. While in Asia all sectors respond in a similar way to the market factor before and during the pandemic, in the US we see more variation across sectors in their market loadings. The difference in the US between the two periods is mostly associated with increases in the tails of the distributions during Covid-19 rather than the average value of the beta. The outlier is companies specializing on industrial real estate which experience an increase in their sensitivity to the market during the pandemic – from close to zero to about 0.5. Panel B shows the factor loadings for the real estate factor (REF). In both regions we see that the distribution of betas widens during the pandemic and the average beta goes up in most sectors in the US and in all sectors in Asia. As documented above, the biggest change in observed for office companies in Asia and for hotels in the US. Table 4 reports the coefficient estimates for the REF betas. The highest beta is for office companies in Asia 0.59 during the pandemic as compared to 0.21 prior to the outbreak. The standard deviation across the companies also almost doubles during the first months of Covid-19. In the US, the highest beta during the pandemic is reported for companies specializing on retail, 1.17, up from 0.97. The standard deviation also increases. The biggest change in the sensitivity to the real estate factor is however observed for hotel companies – from 0.38 prior to the pandemic to 0.74 during the outbreak. For industrial the opposite effect is observed, betas decline during Covid-19 as does the standard deviation across the firm betas.

**Table 4:** Real estate factor betas pre-pandemic and during Covid-19

	mean	sd
Panel A: Asia Developed		
Diversified		
REF beta (P)	0.24	0.39
REF beta (C)	0.37	0.34
Hotel		
REF beta (P)	0.24	0.20
REF beta (C)	0.48	0.46
Industrial		
REF beta (P)	0.23	0.14
REF beta (C)	0.43	0.28
Office		
REF beta (P)	0.21	0.18
REF beta (C)	0.59	0.37
Other		
REF beta (P)	0.28	0.28
REF beta (C)	0.56	0.41
Retail		
REF beta (P)	0.25	0.26
REF beta (C)	0.48	0.46
Total		
REF beta (P)	0.24	0.32
REF beta (C)	0.43	0.37
Observations	195	
Panel B: US		
Diversified		
REF beta (P)	0.49	0.49
REF beta (C)	0.78	0.58
Hotel		
REF beta (P)	0.38	0.45
REF beta (C)	0.74	0.48
Industrial		
REF beta (P)	0.80	0.47
REF beta (C)	0.77	0.29
Office		
REF beta (P)	0.85	0.26
REF beta (C)	0.89	0.42
Other		
REF beta (P)	0.81	0.44
REF beta (C)	0.88	0.58
Retail		
REF beta (P)	0.97	0.40
REF beta (C)	1.17	0.64
Total		
REF beta (P)	0.73	0.47
REF beta (C)	0.90	0.57

The differences across sectors are again more pronounced in the US. The dispersion of risk loadings in the retail and hotel sectors in the US increases substantially in the Covid-19 period while it does not change for industrial. Industrial companies even experience a lower average REF loading. In Panel C, I show the distribution of the alphas by sector. The lowest negative alpha is observed for office companies in Asia and for retail and hotel in the US in the pandemic. The non-market returns decrease for all sectors and the distribution widens, experiencing fat tails.

Finally, in Panel D, we show the factor loadings of the Covid Risk Factor (CRF). In the Asian companies, the difference between the sectors in small with the office companies having the largest betas of 0.69, as reported in Table 5. All sectors have positive loadings. In the US, there are large differences across the sectors with retail, hotel and diversified having positive betas whereas the remaining sectors having negative betas. The highest CRF loading is for hotels and is 1.49 (see Table 5), followed by retail (0.95). Industrial and office have negative CRF betas. This means that those sectors respond countercyclical to companies with high Covid-19 exposure.

**Table 5:** CRF beta summary statistics

	mean	sd	p5	p25	p50	p75	p95
Panel A: Asia Developed							
Diversified	0.40	0.56	−0.66	0.11	0.45	0.74	1.21
Hotel	0.52	0.84	−1.05	0.11	0.51	0.95	2.00
Industrial	0.43	0.53	−0.92	0.12	0.40	0.81	1.27
Office	0.69	0.70	−0.12	0.24	0.65	1.14	1.89
Other	0.61	0.38	−0.02	0.36	0.53	0.86	1.46
Retail	0.49	0.56	−0.37	0.12	0.39	0.85	1.61
Total	0.47	0.59	−0.66	0.14	0.48	0.85	1.35
Panel B: US							
Diversified	0.41	0.93	−1.18	−0.16	0.14	1.08	2.05
Hotel	1.49	1.42	−0.21	0.55	1.35	2.11	3.09
Industrial	−0.30	0.63	−1.09	−0.67	−0.52	0.12	1.33
Office	−0.03	0.53	−0.64	−0.35	−0.10	0.12	0.49
Other	0.13	0.76	−0.90	−0.37	−0.07	0.53	1.92
Retail	0.90	0.96	−0.39	0.20	0.78	1.42	2.61
Total	0.46	1.05	−0.83	−0.27	0.2	1.06	2.24

### Fama–MacBeth Regressions

In the second stage, I report the estimated from Fama–MacBeth regressions. Table 6 shows the baseline Fama–MacBeth regressions for three sub-periods – for the per-Covid period, for the Covid-19 period starting in January (Model 2) and for the Covid-29 period starting in February (Model 3 – Covid 2). The betas are calculated in the previous step using Eq. (2). I include the idiosyncratic volatility following Ang et al. (2009). It measures how past firm idiosyncratic volatility can explain returns as it has previously been shown that this effect is significant is some cases. Past idiosyncratic volatility is the standard deviation of the residual of a factor model on the MSCI World and the FTSE EPRA/NAREIT global index returns for a period of one month prior to the respective sample period. This follows the calculation in Ang et al. (2009).

**Table 6:** Baseline Fama–MacBeth regressions before and during Covid-19 outbreak

	(1) Pre	(2) Covid	(3) Covid 2
Panel A: Asia Developed			
MF beta	−0.0006 [−0.0004]	−0.0041*** [−0.001]	−0.0009 [−0.0015]
Mkt-RF (Japan)	−0.0007* [−0.0004]	−0.0017 [−0.0013]	−0.0015 [−0.0018]
SMB beta	0.0003 [−0.0003]	−0.0001 [−0.0004]	0.0008 [−0.0006]
HML beta	−0.0003 [−0.0003]	0.0022*** [−0.0004]	0.0027*** [−0.0006]
REF beta	−0.0002 [−0.0005]	−0.0060*** [−0.0013]	−0.0025 [−0.0019]
resid SD	0.0731* [−0.0425]	0.0183 [−0.0339]	0.0064 [−0.0668]
CRF beta			−0.0005 [−0.0008]
TotalAssets_ln	0 [−0.0001]	0.0004*** [−0.0002]	0.0005** [−0.0002]
DtA	0.0002 [−0.0012]	0.003 [−0.0018]	0.0045 [−0.003]
PtB	−0.0001 [−0.0004]	0 [−0.0006]	−0.0009 [−0.0008]
ROAA	0.0033 [−0.0047]	−0.0055 [−0.0055]	−0.0119 [−0.0087]
REIT	−0.0008*** [−0.0003]	0.0012** [−0.0006]	0.0017** [−0.0008]
Hong Kong	−0.0004 [−0.0006]	0.0001 [−0.001]	0.0019 [−0.0015]
Singapore	0.0004 [−0.0004]	0.0016** [−0.0007]	0.0044*** [−0.001]
Intercept	0 [−0.0013]	−0.0109*** [−0.0027]	−0.0135*** [−0.0039]
R-squared	0.238	0.666	0.692
N	165	165	165
Panel B: US			
MF beta	−0.0002 [−0.0005]	−0.0035*** [−0.0011]	−0.0050*** [−0.0018]
SMB beta	0.0003 [−0.0003]	−0.0008*** [−0.0003]	−0.0014** [−0.0006]
HML beta	−0.0009 [−0.0006]	−0.0036*** [−0.0005]	−0.0045*** [−0.0008]
REF beta	−0.0016** [−0.0006]	−0.0065*** [−0.0012]	−0.0101*** [−0.0017]
resid SD	0.0122 [−0.0309]	−0.0815 [−0.0933]	−0.092 [−0.0898]
CRF beta			−0.0028*** [−0.0006]
TotalAssets_ln	0.0001 [−0.0001]	−0.0001 [−0.0003]	0.0001 [−0.0004]
DtA	0.0007 [−0.001]	−0.0015 [−0.0025]	−0.0035 [−0.0037]
PtB	0 [−0.0001]	0.0003 [−0.0003]	0.0008* [−0.0005]
ROAA	0.0135*** [−0.0049]	0.0223** [−0.0108]	0.0312** [−0.0156]
REIT	0 [−0.0006]	0.0021** [−0.001]	0.0025 [−0.0018]
Intercept	−0.0011 [−0.0018]	−0.0004 [−0.0048]	−0.0037 [−0.0053]
R-squared	0.433	0.78	0.763
N	178	178	178

MF beta stays for the beta of the region specific Fama French market factor. SMB beta stays for the beta of the region-specific Fama French size factor. HML beta stays for the beta of the region-specific Fama French value factor. REF beta stays for the beta of the region-specific FTSE-EPRA-NAREIT real estate factor. DtA stays for the debt-to-assets ratio. PtB stays for the price-to-book ratio. ROAA stays for the return on assets ratio, REIT is a dummy that takes 1 if the company has a real estate investment trust status. Hong Kong and Singapore are country dummies and the regressions are run using Japan as a reference point. All betas and alphas in models (1) and (2) are estimated using the factor model in Eq. (2). Betas and alphas in Model (3) are estimated using Eq. (2) including a the Covid Risk Factor (CRF). Model (1)’s inputs are based on a Fama French model estimated from 1 November 2019 until 22 January 2020. The residual standard deviation (resid SD) or the past idiosyncratic risk is the SD of the lagged residual estimated for the period of 1 October 2019 until 31 October 2019. Model (2)’s inputs are based on a Fama French model estimated from 24 January 2020 until 21 April 2020. The residual standard deviation (resid SD) is the SD of the lagged residual estimated for the period of 20 December 2019 until 22 January 2020. All regressions are conducted with sector fixed effects and standard errors are clustered by firm ID. Standard errors are reported under each coefficient. ***, **, * stay for 1%, 5% and 10% significance levels respectively

In addition, I control for company-specific financial information which may affect return performance in the cross-section. This includes controls for size effects using total asset capitalisation (TotalAssets_ln), leverage constraints using the debt-to-asset (DtA) ratio, valuation effects using the price-to-book (PtB) ratio, profitability effects using the return on average assets (ROAA). I also include a dummy that captures whether the real estate company has a REIT status (REIT). All regressions are conducted with sector and country fixed effects (for the Asian sub-sample) and standard errors are clustered by firm ID.

The primary focus is on the cross-sectional relationship between risk – both systematic and idiosyncratic – and return. Table 6 shows the results for Asia and the US separately. The dependence between average returns across firms and MF betas is not significant prior to the pandemic and becomes significantly negative during the pandemic in both regions. The model fit also improves substantially during the pandemic. In Asia the R-squared goes up from 0.238 to 0.666 (Model 2) and in the US – from 0.433 to 0.78. The effect of the value factor loadings also turns from insignificant prior to Covid-19 to significant during the first months of the pandemic. Although the sign in from of the HML beta is positive in China, the betas are negative which means that the overall effect has the same interpretation as the one in the US.

While one would expect an upward sloping security market line (SML) – a positive relationship between returns and risks (beta) – the opposite is the case. The fact that market risk is associated negatively with firm returns and abnormal returns means that the higher the risk the lower the return. This negative relationship is also known as the low-risk effect – stocks with low risk have high returns.

When we include the CRF loading in the models (i.e. Model 3 in Table 6), the results remain similar. The CRF beta has a significantly negative effect on returns in the US whereas it is not significant in Asian firms. Returns in the US are also explained by their exposure to the coronavirus risk in addition to other systematic risks. The higher the exposure to the systemic risk, the lower the return of a firm would be. This can be due to sentiment drivers which can be tested by including idiosyncratic risk in the Fama Macbeth models. However, residual variation has no significant effect in any of the regions and sub-periods.

Cross-sectional returns do not seem to be driven by firm-specific financials apart from firm size and REIT status in Asia. In the US, ROAA has a significantly positive effect on returns in the cross-section. Market participants seem to already incorporate information about firm fundamentals in the returns, both before and during the pandemic. The main difference between the two periods stems from the market risk. In particular, the value risk factor loading is significant in the Covid-19 period for the two specifications, both in Asia and the US, suggesting that differences in valuations may play for cross-sectional performance during the pandemic.

Table 7 shows the sector fixed effects of the Fama MacBeth models. The baseline sector is Diversified against all other sectors are compared. In the US, there are no significant differences across the sectors prior to the pandemic with the only exception of Casinos which outperform Diversified. During the pandemic, firms specializing on regional malls and shopping centers perform significantly worse than companies with diversified assets. On the other hand, Speciality performs significantly better as compared to Diversified. As demonstrated in Ling et al. (2020), sector specialisation in real estate holdings does play an important role for company performance.

**Table 7:** Sector fixed effects

	(1) Pre	(2) Covid	(3) Covid 2
Panel A: Asia Developed			
Health Care	−0.0006 [−0.0004]	−0.0002 [−0.0011]	0.0003 [−0.0013]
Hotel	0 [−0.0004]	0.001 [−0.0009]	0.0013 [−0.0012]
Industrial	0 [−0.0003]	0.0012 [−0.0008]	0.0016 [−0.0011]
Multifamily	−0.0003 [−0.0006]	0 [−0.0009]	0.0011 [−0.0011]
Office	0 [−0.0003]	−0.0003 [−0.0005]	−0.0008 [−0.0008]
Other Retail	−0.0007* [−0.0004]	0.0003 [−0.0011]	−0.0004 [−0.0011]
Regional Mall	−0.0002 [−0.0002]	−0.001 [−0.0007]	−0.0017 [−0.0012]
Shopping Center	−0.0006** [−0.0003]	0.0009 [−0.0011]	0.0019 [−0.0016]
Specialty	0.0001 [−0.0003]	0.0019*** [−0.0005]	0.0047*** [−0.0011]
Panel B: US			
Casino	0.0013*** [−0.0004]	0.0008 [−0.0018]	0.0018 [−0.0031]
Health Care	−0.0001 [−0.0006]	0.0013 [−0.001]	0.0019 [−0.0014]
Hotel	−0.0006 [−0.0006]	−0.0014 [−0.0011]	−0.0002 [−0.0018]
Industrial	−0.0002 [−0.0005]	0.0015 [−0.0012]	0.0022 [−0.0016]
Manufact. Home	0.0001 [−0.001]	0.0017 [−0.0013]	0.0012 [−0.0019]
Multifamily	−0.0008 [−0.0005]	0.0006 [−0.001]	0.0005 [−0.0017]
Office	0.0006 [−0.0005]	0.0002 [−0.001]	0.0016 [−0.0014]
Other Retail	−0.0004 [−0.0005]	−0.0001 [−0.0014]	0.0003 [−0.0019]
Regional Mall	−0.0007 [−0.0011]	−0.0085*** [−0.0026]	−0.0048* [−0.0029]
Self-Storage	−0.0005 [−0.0005]	−0.001 [−0.0013]	−0.0018 [−0.0018]
Shopping Center	−0.0008 [−0.0005]	−0.0045*** [−0.0014]	−0.0054** [−0.0021]
Specialty	0.0001 [−0.0006]	0.0030** [−0.0013]	0.0039** [−0.0016]

The results are a continuation of the results in the previous table. The dependent variable is daily returns. The baseline sector is Diversified against which all other sectors are compared. Standard errors are reported under each coefficient. ***, **, * stay for 1%, 5% and 10% significance levels respectively. Modell (1) is estimated prior to the pandemic. Model (2) is estimated during the pandemic. Model (3) is estimated on a shorter sample during the pandemic. See Notes from the previous table

In Asia, shopping centers and other retail performed worse prior to the pandemic. During the early stages of Covid-19, however, the difference across sectors vanishes away (with the exception of Specialty, which outperforms), which is in line with the observations from the descriptive statistics and factor model regressions.

To sum up, while the pandemic leads to significant divergence in the performance of companies depending on the real estate sector they specialize on in the US, the opposite trend is observed in Asian real estate companies.

### Robustness Tests

Instead of using the Fama French factors, I estimate all factor models using the stock market factors and the real estate factor instead. Overall, the key results remain robust. Furthermore, in addition to using a Covid-19 risk factor, I estimate the factor models in Eq. (2) directly using Covid-19 cases data. In one of the robustness I use the daily change in confirmed cases as the measure for exposure to infection. In another specification I use the natural logarithm of the Covid-19 cases. The results remain robust.

## Conclusion

This paper explores changes to the cross-sectional risk-return relationship as a result of Covid-19 and in two regions – Asia and the US. In addition, I construct a Covid Risk Factor (CRF) to assess the sensitivity of individual firms to Covid-19 risks. The risk factor is linked to daily changes in confirmed global Covid-19 cases and is not correlated with other stock market factors. The research examines how Covid-related exposure affecta the cross-section of returns in addition to other systematic and idiosyncratic risks. Following on from the recent research by Ling et al. (2020) on the effects of Covid-19 on REIT returns, this study is also among the first to assess asset real estate stock market pricing behaviour as a result of Covid-19 internationally.

There are a number of findings. First, the returns of real estate companies experience a sharp decline and a fat-tailed distribution as a result of Covid-19 with large differences across sectors in the US. Second, there are considerable differences in the response of Asian companies as compared to US companies. Although the pandemic originated in China and first spread there before the virus outbreak became a global phenomenon, returns of Asian-based companies were less negatively affected as compared to those in the US. Furthermore, the two regions show strong sector-based divergence in performance as a result of their response to Covid-19. While US real estate companies show strong differences in performance based on the real estate sector they specialize on, little sectoral variation is observed in the Asian region. The sector with significant underperformance during the pandemic is retail in the US. I also assess the effect of Covid-19 risks on returns during the pandemic. While in Asia companies do not vary in their sensitivity to Covid risks across sectors, in the US we see large differences. The factor models incorporating the CRF show that hotel has the highest sensitivity to Covid-19 risks, while in Asia and it is the office sector.

The sensitivity of firms during the Covid-19 period increases for most risk factors in the US. In Asia, I observe that the sensitivity prior to the pandemic is close to zero and becomes largely negative during the first few months of the coronavirus. This suggests that Asian and US firms have very different response to the pandemic which may be partially due to the experience with similar coronaviruses in Asia. Asian real estate companies can provide a good hedge during similar periods of global economic shocks, although their non-market performance is significantly negatively affected.

Finally, Fama–MacBeth regressions show that the main effect of Covid-19 for the cross-section of returns is associated with market risk factors. Again, Asia and US differ in this regard although what is common for both regions is the significant role played by the value factor risk loading indicative of valuation effects dominating during the Covid-19 period. In the US, companies show significantly positive response to the CRF sensitivity indicative while this is not the case in Asia. The relationship between the market factor loadings and returns is positive which points towards a low-risk effect triggered by Covid-19 but not related to sentiment as documented by the insignificant coefficient of the idiosyncratic volatility.

## Footnotes

1 The anticipated R, or infection rate, during the peak of the virus is between 3 and 4 and varies by time and location.

2 However, the mortality rate is expected to be about ten times higher than that of a normal flu.

3 The US has followed with a large number of states adopting some form of a lockdown in March and April 2020.

4 The financial variables are available in March 2020 and refer to the previous financial year, therefore they do not reflect the effect of the pandemic yet.
